# The Impact of the Nucleosome Code on Protein-Coding Sequence Evolution in Yeast

**DOI:** 10.1371/journal.pgen.1000250

**Published:** 2008-11-07

**Authors:** Tobias Warnecke, Nizar N. Batada, Laurence D. Hurst

**Affiliations:** Department of Biology and Biochemistry, University of Bath, Bath, United Kingdom; Stanford University, United States of America

## Abstract

Coding sequence evolution was once thought to be the result of selection on optimal protein function alone. Selection can, however, also act at the RNA level, for example, to facilitate rapid translation or ensure correct splicing. Here, we ask whether the way DNA works also imposes constraints on coding sequence evolution. We identify nucleosome positioning as a likely candidate to set up such a DNA-level selective regime and use high-resolution microarray data in yeast to compare the evolution of coding sequence bound to or free from nucleosomes. Controlling for gene expression and intra-gene location, we find a nucleosome-free “linker” sequence to evolve on average 5–6% slower at synonymous sites. A reduced rate of evolution in linker is especially evident at the 5′ end of genes, where the effect extends to non-synonymous substitution rates. This is consistent with regular nucleosome architecture in this region being important in the context of gene expression control. As predicted, codons likely to generate a sequence unfavourable to nucleosome formation are enriched in linker sequence. Amino acid content is likewise skewed as a function of nucleosome occupancy. We conclude that selection operating on DNA to maintain correct positioning of nucleosomes impacts codon choice, amino acid choice, and synonymous and non-synonymous rates of evolution in coding sequence. The results support the exclusion model for nucleosome positioning and provide an alternative interpretation for runs of rare codons. As the intimate association of histones and DNA is a universal characteristic of genic sequence in eukaryotes, selection on coding sequence composition imposed by nucleosome positioning should be phylogenetically widespread.

## Author Summary

Why do some parts of genes evolve slower than others? How can we account for the amino acid make-up of different parts of a protein? Answers to these questions are usually framed by reference to what the protein does and how it does it. This framework is, however, naïve. We now know that selection can act also on mRNA, for example, to ensure introns are removed properly. Here, we provide the first evidence that the way DNA works also affects gene and protein evolution. In living cells, most DNA wraps around histone protein structures to form nucleosomes, the basic building blocks of chromatin. Protein-coding sequence is no exception. Looking at genes in baker's yeast, we find that sequence between nucleosomes, linker sequence, is slow evolving. Both mutations that change the gene but not the protein and those that change gene and protein are affected. We argue that selection for correct nucleosome positioning, rather than differences in mutational processes, can explain this observation. Linker also exhibits distinct patterns of codon and amino acid usage, which reflect that DNA of linker needs to be rigid to prevent nucleosome formation. These results show that the way DNA works impacts on how genes evolve.

## Introduction

In simple models of molecular evolution, selection on protein coding sequence (CDS) is exclusively devoted to optimizating protein function. As such, we expect amino acid choice to be dictated by protein function alone and synonymous mutations to be neutrally evolving. This is now known to be naïve. The protein's mRNA template can be under selection to maintain favourable mRNA structure [1]–[5] or facilitate speedy and accurate translation through usage of certain synonymous codons [6]–[10]. There is also evidence for selection on regulatory motifs in exons required for correct splicing [11]–[14]. Thus, many stages of the protein production chain are subject to their own particular regimes of selective constraint. But is this also the case when protein-coding information is still stored as DNA in its chromosomal context? In other words, does the way DNA is organized come with its own important requirements on sequence composition, requirements that potentially conflict with optimization of protein function or translation rate optimization or any of the other forces?

One candidate process that might set up selective constraint at the DNA level is nucleosome positioning. Nucleosomes are the elementary units of chromatin organization, at their core comprising a ∼147 bp stretch of DNA tightly wrapped around a histone protein octamer. These core parcels are separated along the chromosome by “linker” regions of variable length [15]. At least two aspects of nucleosome architecture combine to make effects on coding sequence evolution a distinct possibility. First, the histone core has characteristic DNA-binding preferences [16]–[18], governed by the variable bending and twisting attributes of different sequences [19]. Although nucleosomes can form on any stretch of DNA [15], relative affinities can differ by several orders of magnitude [20]. In consequence, nucleosome positioning partly reflects the equilibrium state expected under a model in which energy penalties for coercing rigid DNA into a nucleosome state are minimized [21]. For example, nucleosome-free regions are enriched in rigid poly-A and poly-T runs [22],[23]. Second, selection is likely to favour nucleosomes to be present at particular intra-genic sites and not at others. In particular, well-positioned nucleosomes frequently flank transcriptional start sites thus determining promoter accessibility [23]–[26]. Given that nucleosome formation preferentially occurs on particular sequences, but positioning cannot be entirely opportunistic because it is oriented relative to functional motifs, we might expect coding sequence composition to be biased and its evolution to be constrained to maintain adequate nucleosome architecture.

To examine this expectation we make use of a recent high-resolution (4 bp) genome-wide nucleosome map for *Saccharomyces cerevisiae* [23]. Based on evidence from codon and amino acid usage as well as comparative rates of evolution we identify nucleosome positioning as a novel layer of selection acting on protein-coding DNA.

## Results

### Nucleosome Occupancy Covaries with Expression

Based on the experimentally determined *S. cerevisiae* nucleosome map of Lee and colleagues [23], we assigned a likely occupancy state (OS) to each coding nucleotide. OSs comprise putatively unoccupied linker region, fuzzily positioned nucleosomes, and well-positioned nucleosomes (see Methods). For intra-specific comparison of compositional differences, genes were then “abridged” so that they only contained codons that were predicted to have the same OS (see Methods). Assuming that occupancy is relatively static over the evolutionary time scale analyzed here, we can also study differences in sequence evolution as a function of OS. *S. cerevisiae* codons from abridged genes that could be assigned to an orthologous codon in *S. mikatae* were retained for inter-specific comparison. Results of all orthology-based analyses are largely insensitive to choice of close comparator species, with *S. bayanus* or *S. paradoxus* orthologues showing the same trends (data not shown).

Analyzing evolutionary rates solely as a function of nucleosome occupancy is likely to yield misleading results because covariates common to both nucleosome architecture and sequence evolution are not controlled for. Prominently, selection on translational accuracy, speed, and robustness requires attention. Translational selection has been put forward as the single most important cause of between-gene variation in evolutionary rates in yeast [27], where highly expressed genes show reduced rates of non-synonymous [28] and synonymous [27] substitutions as well as substantial codon bias [29]. More acutely, expression intensity is linked to promoter-type [30], which in turn is linked to where, and how, nucleosomes are positioned. Nucleosomes tend to be depleted from promoters [24],[25],[31] but enriched over the coding regions [23] of highly expressed genes. In fact, Shivaswamy and colleagues [26] recently demonstrated that poorly positioned, i.e. fuzzy, nucleosomes over the CDS are associated with high transcription rates.

Considering genes (N = 1718) for which information is available on evolutionary rates, nucleosome occupancy and protein abundance [32], we confirm proportional OS composition as a quantitative marker of expression (Kendall's tau (%linker∼abundance) = −0.24, P≪0.0001; tau (%fuzzy∼abundance) = 0.11, P<0.0001; tau (%wp∼abundance) = −0.07, P<0.0001). Protein abundance is the expectedly strong negative predictor of evolutionary rates (Spearman's rho (abundance∼K_a_) = −0.47, P<0.0001; rho (abundance∼K_s_) = −0.38, P<0.0001) linking OS composition to K_s_ (rho (%fuzzy∼K_s_) = −0.06, P<0.0001) and, more pertinently, K_a_ (rho (%fuzzy∼K_a_) = −0.1, P<0.0001). Consequently, controlling for expression in analyzing the impact of nucleosome occupancy is imperative.

### Within a Gene, Linker Sequence Evolves Slowest

The ideal approach to eliminate differences in expression between genes is to compare OS-linked evolution within genes. Within-gene analysis suggests that linker sequence exhibits reduced synonymous and non-synonymous evolution (ΔK_a_(well-positioned v linker): 15%, paired t-test: 4.37, P<0.0001; ΔK_a_(fuzzy v linker): 7%, paired t-test: 1.61, P<0.11; ΔK_s_ (well-positioned-linker): 10%, paired t-test: 4.64, P<0.0001; ΔK_s_ (fuzzy-linker): 12%, paired t-test: 5.47, P<0.0001; N = 158; see Methods). These results offer preliminary support for the hypothesis that linker sequence is under stronger purifying selection than non-linker sequence at both synonymous and non-synonymous sites.

### Intra-Gene Position Needs to be Taken into Account

**Figure 1:**
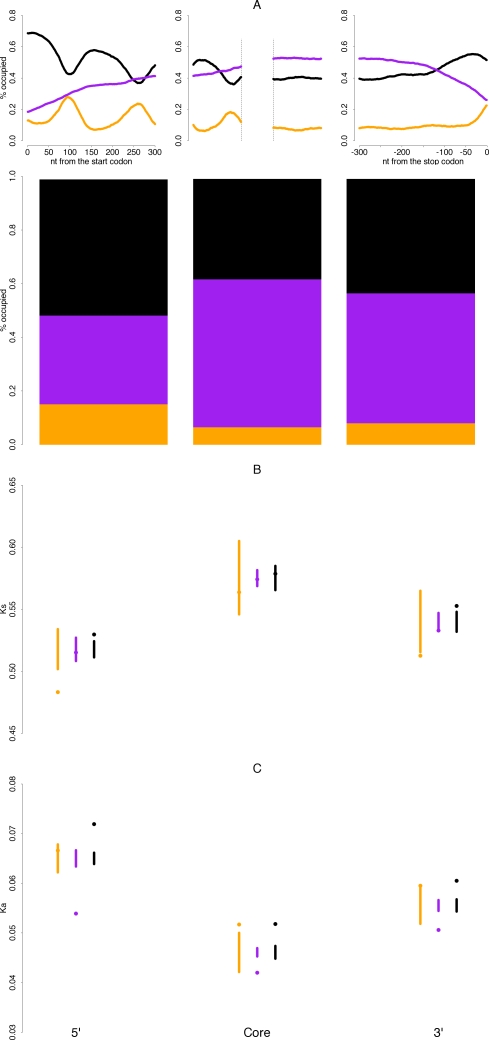
Regional biases in nucleosome occupancy. (A) Occupancy states are unevenly represented across CDS regions. The top panel shows regional variation in the proportion of linker (orange), fuzzy (purple), and well-positioned (black) nucleosomes across yeast CDS regions. In the core panel, the 150 codons bordering each CDS end are depicted. The bottom panel gives mean proportions of nucleotides called as one of the three occupancy states for the terminal 100 codons and the core across genes ≥906 nt. (B, C) CDS regions have distinct substitution dynamics but differences linked to nucleosome occupancy are still evident within regions. Rates of synonymous (B) and non-synonymous (C) evolution between *S. cerevisiae* and *S. mikatae* discriminated by CDS region and occupancy state. The dot represents the respective rate determined from the concatenated sequence. The vertical bar represents the distribution of K_a_(K_s_) values expected under a random model (see Methods) where identity of aligned codons is independent of nucleosome occupancy. Data for the restricted core are shown to make variances comparable.

However, within-gene comparisons can only be carried out for a small number of genes (N = 158) because rarely is there sufficient sequence for all OSs within the same gene to obtain reliable rate estimates. Consequently, this sample is biased towards very long genes (see Methods). Further, within-gene comparisons might still not reflect the true relationship between nucleosome occupancy and sequence evolution if there is intra-genic heterogeneity in substitution dynamics. This is because nucleosomes exhibit promoter-specific architectures, in line with their role in regulating promoter accessibility [23],[25]. As the majority of translational start sites (ATG) in yeast are positioned within one nucleosomal rotation of the transcriptional start site [33], 5′ ends of CDSs show regular occupancy patterns (Figure 1A), which have repeatedly been described in the literature. This intimate association of CDS region and OS only gradually collapses downstream because linker length variation is typically modest [23]. Furthermore, regularities can also be detected across 3′ ends of CDS [26] (Figure 1A). If, then, there existed gene-region distinct evolutionary trajectories, we would expect any analysis of OS-based differences to be biased as a result of the uneven representation of OSs across these regions (Figure 1A bottom panel).

To address the issue of regional biases and increase the amount of available sequence, we chose a concatenation-based approach. Eligible codons were concatenated across all genes ≥906 nt (N = 845) by region (5′, core, 3′) and OS. The terminal 100 codons were taken to represent 5′ and 3′ regions. For the core region, we analyzed the central 100 codons (“restricted core”) as well as all sequence after the termini are removed (see Methods and Table S1). As depicted in Figures 1B&C, there is indeed a marked regional component to coding sequence evolution, with K_s_ reduced at the CDS periphery and K_a_ at the centre of genes. That reduced synonymous substitutions at CDS termini can combine with low amino acid substitutions towards the centre of the gene has been observed previously in bacteria [34]. Selection on translational control mechanisms [35]–[37] and Hill-Robertson effects [38] might be the cause of regionally distinct K_s_ while the explanation for intra-genic variation in K_a_ is more elusive. Whatever the cause, the result is a spatial bias likely to confound analyses of nucleosome-related sequence evolution by inflating existing trends. In particular, linker sequence evolves particularly slowly at 5′ ends, where it is most prevalent (Figure 1A bottom panel). Importantly, however, OS-linked differences are still manifest *within* regions (Figure 1B&C, Table S1). Thus, regional biases are insufficient to explain why sequences show distinct evolutionary patterns depending on OS.

### Controlling for Expression Reveals Lower Rates of Evolution in Linker Sequence

From the described results, a contradictory finding emerges. When comparing evolutionary rates within genes, we found K_a_ and K_s_ both reduced in linker sequence, yet in the regional analysis K_a_ and K_s_, oddly, disagree. K_a_ appears reduced for fuzzy sequence (Figure 1C). This discrepancy, however, might be an artefact of fuzzy sequence being enriched in highly expressed genes, which in turn show elevated levels of amino acid conservation [28].

**Figure 2:**
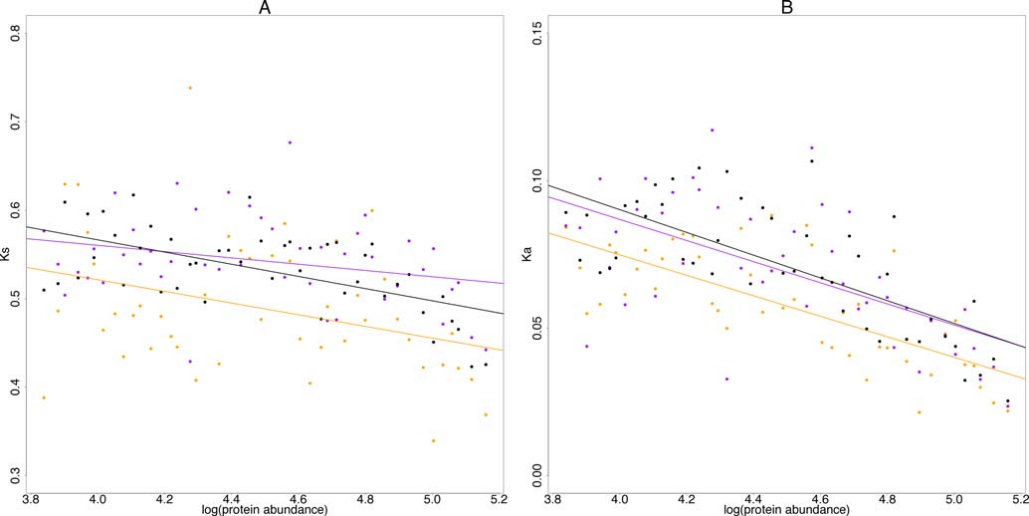
Controlling for protein abundance in the analysis of OS-linked differences in evolutionary rates. (A) Synonymous and (B) nonsynonymous rates of evolution across 5′ ends of genes as a function of both the natural logarithm of protein abundance and nucleosome occupancy (see Methods for details of binning protocol). Regression lines are fitted for individual occupancy states.

To evaluate this possibility, sequence concatenated by region and OS was further binned by protein abundance (see Methods). Although noise is substantial, Figures 2A&B illustrate for 5′ regions that controlling for expression recreates a more consistent picture of substitution dynamics. Synonymous but also non-synonymous substitution rates are reduced in linker regions (Table 1, Methods) by ∼6% (Table S2). K_s_ but not K_a_ is also reduced in core regions (by ∼5%) while we detect no significant differences in substitution rates between OSs across 3′ regions (Table 1). Evolutionary rates of sequence associated with fuzzily or well-positioned nucleosomes are virtually indistinguishable (Table S2). Thus, the reduced K_a_ for fuzzy sequence observed in Figure 1C is an artifact of the enrichment of fuzzy sequence in highly expressed genes.

**Table 1:** Analysis of covariance testing whether rates of evolution (*S. cerevisiae* – *S. mikatae*) group by occupancy state when region and protein abundance are controlled for.

	CDS region	All available codons					Codons matched across occupancy states				
		Median rate across bins			ANCOVA (F)	P	Median rate across bins			ANCOVA (F)	P
		L	F	WP			L	F	WP		
Ks	5′	0.477	0.539	0.535	12.1	**1.51E-05**	0.477	0.544	0.544	9.82	**0.0001**
	Core	0.563	0.608	0.617	12.14	**1.38E-05**	0.563	0.603	0.607	5.6	**0.005**
	3′	0.517	0.564	0.564	1.07	0.35	0.517	0.564	0.566	1.01	0.37
Ka	5′	0.057	0.068	0.071	9.62	**0.0001**	0.057	0.068	0.072	4.75	**0.01**
	Core	0.054	0.06	0.058	1.7	0.19	0.054	0.063	0.056	2.39	0.1
	3′	0.067	0.064	0.074	1.02	0.37	0.067	0.054	0.077	0.98	0.38

Data for both all available codons and codons matched across occupancy states (see main text) are shown. L, F, and WP stand for sequence associated with linker, fuzzily and well-positioned nucleosomes, respectively.

Patterns of single nucleotide polymorphisms (SNPs) suggests that whichever factors have caused OS-linked differences in divergence are still a relevant evolutionary force in current populations of *S. cerevisiae*. Analyzing polymorphism data from a recent re-sequencing effort of over 30 *S. cerevisiae* strains (see Methods), we found SNP density in the same set of genes to be reduced relative to random expectation at synonymous (chi-square test = 35.61, P = 1.8E-08, enrichment: linker: 0.89, fuzzy: 1.00, well-positioned: 1.02) and non-synonymous sites (chi-square test = 11.48, P = 0.0032, enrichment: linker: 0.95, fuzzy: 1.04, well-positioned: 0.98). These trends become even more clear-cut when expression is controlled for (data not shown).

### Mutational Bias Does Not Explain Why Codons Preferred in Linker Evolve More Slowly

Although the above results support the notion that purifying selection is stronger in linker than in non-linker, this need not be the correct interpretation. Linker sequence might simply be less mutable. This could be for one of two reasons. First, codons enriched in linker are less mutagenic. Second, regardless of codon composition, linker is somehow protected from mutation.

As regards the first possibility, codons preferentially employed in linker sequence are noticeably AT-rich (see below). As G and C are typically considered more mutable, this alone may explain low evolutionary rates in linker. We control for this scenario in the following way: for every aligned *S. cerevisiae* linker codon, we randomly select (without replacement) an identical *S. cerevisiae* codon from the pool of identical codons in the fuzzy and well-positioned concatenated sequences in the same expression/region bin respectively. In the small number of cases where a linker codon could not be matched to a codon in a different OS, a codon was chosen at random. In this way, we end up with sequences of the same length as the linker sequence and virtually identical codon composition. Table 1 reveals that, controlling for codon composition, we find the same pattern of constraints uncovered previously (also see Table S2). We conclude that the low rates of evolution observed for linker sequence are not more parsimoniously explained by an AT-mutation bias.

Could it be that linker sequence is less mutagenic, regardless of codon content? One can imagine mechanistic models in which this might be possible. For example, Kepper et al. [39] recently explored the links between chromatin fiber conformation and nucleosome geometry. Their models, based on mammalian chromatin, suggest that during higher-order organization of nucleosomes into compact chromatin fibers linker sequence is brought into the core of the chromatin fiber upon binding of linker histone, and might be better protected against mutagens as a result. It has also been shown that the binding of linker histone Hho1p inhibits homologous recombination [40]. As homologous recombination in yeast is thought to be mutagenic [41]–[43], reduced rates of substitution might be linked to the protective effects of Hho1p binding.

Aside from the fact that it is unclear whether yeast chromatin is organized in a mammal-like fashion as far as higher order structure is concerned, it seems unlikely that mutational effects can be the sole explanation, not least because linker sequence shows different rates of evolution as a function of intra-gene position even when overall regional biases are taken into account. The proportional reduction of linker *K_s_* to synonymous rates of nucleosome-bound sequence in the same bin tends to be significantly higher at 5′ (median reduction = 0.114) versus 3′ ends (median reduction = 0.026, Wilcoxon test P = 0.04), with the difference to core regions not quite significant (median reduction = 0.057, P = 0.07).

### Impact of Nucleosome Code on Codon and Amino Acid Content

**Figure 3:**
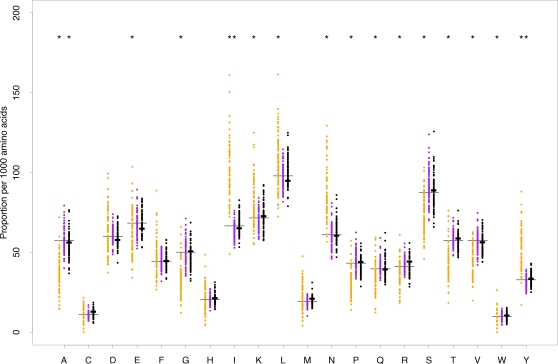
Nucleosome-free regions show a divergent pattern of amino acid usage. Amino acid usage by occupancy state in concatenated CDS cores are shown. Each data point represents an expression bin (see main text). Long and short horizontal bars represent the proportional usage (out of 1000) of the respective amino acid across all amino acids in the current sample and the genome, respectively, regardless of occupancy state. Significant depletion/enrichment relative to the proportional usage across occupancy states in the sample is indicated by an asterisk (Wilcoxon test; significance threshold adjusted to account for multiple testing across occupancy states (3) and amino acids (20), P<8.3E-04). See Table S3 for P values for all regions and amino acids.

If nucleosome positioning is responsible for elevated linker conservation then we might additionally expect to see skews in patterns of codon and amino acid usage. We compared codon and amino acid composition between OSs within the *S. cerevisiae* genome. As alignability is not an issue in this analysis, we can exploit a substantially larger number of genes ≥906 nt (N = 1986). Figure 3 shows for core sequence binned by protein abundance that multiple amino acids are depleted or enriched in linker sequence relative to their proportional use across all core sequence (regardless of OS).

These skews appear linked to nucleosome occupancy. First, some amino acids are coded exclusively by nucleotide trimers that are unanimously, albeit sometimes weakly, predictive of either nucleosome binding or exclusion as determined by Peckham and colleagues for genomic sequence [44] (Table 2). If nucleosome positioning was a relevant functional concern, such amino acids should be depleted from linker sequence if all their codons have a positive positioning score, and vice versa, because they have no capacity to negotiate this concern by adjusting their codon usage. This is what we observe. Eight out of eleven amino acids with unanimous positioning score across all codons show skewed usage in the expected direction (Table 2, Table S3), while the remaining three show no significant skews.

**Table 2:** Amino acid and codon usage biases by CDS region, their relationship to nucleosome positioning attributes, and optimal codon identity.

Amino acid	Codon*	Optimal codon**	Triplet ROC score***	Unambiguous ROC score towards active positioning (P) or exclusion (E) ****	Amino acid significantly depleted (P) or enriched (E) in linker*****			Number of bins in which codon was found to be significantly enriched;depleted in linker sequence		
					5′	Core	3′	5′ (64 bins)	Core (73 bins)	3′ (32 bins)
**A**	GCA		**+0.647774**	**P**	**P**	**P**	**P**	**0;1**	**2;4**	**2;1**
	GCC	**X**	**+0.693062**					**1;0**	**1;5**	**0;3**
	GCG		**+0.644435**					**1;0**	**5;1**	**3;0**
	GCT	**X**	**+0.679455**					**0;1**	**3;3**	**1;2**
**C**	TGC		**+0.647774**					**0;0**	**0;1**	**1;0**
	TGT	**X**	**−0.572361**					**0;0**	**1;0**	**0;1**
**D**	GAC†	**X^d^**	**+0.667029**	**P**				**0;6**	**0;9**	**0;3**
	GAT†		**+0.521952**					**6;0**	**9;0**	**3;0**
**E**	GAA	**X**	**−0.522684**			**P**		**3;0**	**2;0**	**2;0**
	GAG		**+0.657054**					**0;3**	**0;2**	**0;2**
**F**	TTC	**X**	**−0.522684**	**E**				**1;4**	**2;6**	**0;0**
	TTT		**−0.801516**					**4;1**	**6;2**	**0;0**
**G**	GGA		**+0.664171**	**P**	**P**	**P**	**P**	**2;2**	**0;3**	**3;2**
	GGC		**+0.693062**					**2;2**	**2;1**	**2;3**
	GGG		**+0.608406**					**2;2**	**2;1**	**2;3**
	GGT	**X**	**+0.664812**					**2;2**	**0;3**	**3;2**
**H**	CAC	**X**	**+0.646195**					**0;2**	**1;2**	**0;0**
	CAT		**−0.511575**					**2;0**	**2;1**	**0;0**
**I**	ATA†		**−0.810542**		**E**	**E**	**E**	**4;0**	**15;0**	**7;0**
	ATC†	**X^d^**	**+0.521952**					**0;4**	**0;15**	**1;6**
	ATT	**X**	**−0.769963**					**4;0**	**4;11**	**3;4**
**K**	AAA†		**−0.801516**	**E**	**E**	**E**		**7;0**	**8;1**	**1;0**
	AAG†	**X^d^**	**−0.509962**					**0;7**	**1;8**	**0;1**
**L**	CTA		**−0.581168**			**E**	**E**	**6;2**	**9;7**	**0;4**
	CTC		**+0.657054**					**2;6**	**6;10**	**2;2**
	CTG		**+0.707122**					**0;8**	**0;16**	**3;1**
	CTT†		**−0.509962**					**2;6**	**2;14**	**2;2**
	TTA†		**−0.805062**					**8;0**	**16;0**	**4;0**
	TTG†	**X^d^**	**+0.549621**					**1;7**	**2;14**	**0;4**
**M**	ATG	**NA**	**−0.511575**	**E**				**NA**	**NA**	**NA**
**N**	AAC†	**X^d^**	**+0.512141**		**E**	**E**	**E**	**0;20**	**0;28**	**0;7**
	AAT†		**−0.769963**					**20;0**	**28;0**	**7;0**
**P**	CCA	**X**	**+0.736594**	**P**		**P**	**P**	**0;2**	**1;0**	**1;2**
	CCC		**+0.608406**					**1;1**	**1;0**	**1;2**
	CCG		**+0.672283**					**2;0**	**0;1**	**3;0**
	CCT		**+0.620874**					**0;2**	**0;1**	**1;2**
**Q**	CAA	**X**	**+0.549621**	**P**	**P**	**P**		**1;2**	**1;1**	**2;0**
	CAG		**+0.707122**					**2;1**	**1;1**	**0;2**
**R**	AGA	**X**	**+0.523704**	**P**		**P**		**2;4**	**1;2**	**0;1**
	AGG		**+0.620874**					**2;4**	**1;2**	**0;1**
	CGA		**+0.576424**					**4;2**	**3;0**	**1;0**
	CGC		**+0.644435**					**4;2**	**1;2**	**1;0**
	CGG		**+0.672283**					**3;3**	**1;2**	**1;0**
	CGT	**X**	**+0.637626**					**2;4**	**2;1**	**1;0**
**S**	AGC		**+0.679455**		**P**		**P**	**2;1**	**2;1**	**2;0**
	AGT		**−0.511514**					**2;1**	**2;1**	**1;1**
	TCA		**+0.536638**					**3;0**	**3;0**	**1;1**
	TCC	**X**	**+0.664171**					**2;1**	**0;3**	**0;2**
	TCG		**+0.576424**					**1;2**	**2;1**	**1;1**
	TCT	**X**	**+0.523704**					**0;3**	**1;2**	**0;2**
**T**	ACA		**−0.572361**			**P**	**P**	**4;1**	**1;1**	**0;0**
	ACC	**X**	**+0.664812**					**1;4**	**1;1**	**0;0**
	ACG		**+0.637626**					**3;2**	**0;2**	**0;0**
	ACT	**X**	**−0.511514**					**2;3**	**1;1**	**0;0**
**V**	GTA		**−0.612226**					**4;2**	**0;2**	**3;1**
	GTC	**X**	**+0.667029**					**2;4**	**1;1**	**3;1**
	GTG		**+0.646195**					**2;4**	**0;2**	**2;2**
	GTT	**X**	**+0.512141**					**5;1**	**2;0**	**1;3**
**W**	TGG	**NA**	**+0.736594**	**P**		**P**	**P**	**NA**	**NA**	**NA**
**Y**	TAC†	**X^d^**	**−0.612226**	**E**	**E**	**E**	**E**	**0;7**	**0;18**	**0;5**
	TAT†		**−0.810542**					**7;0**	**18;0**	**5;0**

*: codons with significant skews (see Methods) marked †.

**: as determined by Kliman et al. (2003) [45]. X^d^: optimal codon significantly depleted in linker in the core region.

***: as determined by Peckham et al. (2007) [44]. Positive score indicates that triplet is predictive of nucleosome binding. See Methods for a brief explanation of the receiver operating characteristic (ROC).

****: i.e., all synonymous codons show either positive or negative ROC scores.

**Figure 4:**
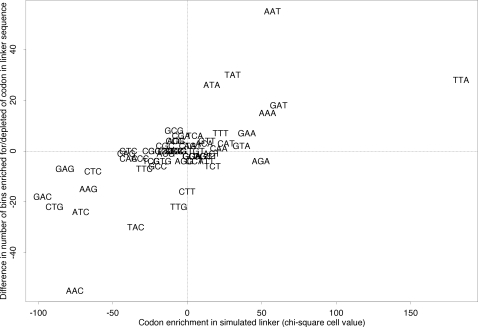
Codon usage in experimentally determined linker reflects nucleosome exclusion potential. Enrichment/depletion of synonymous codons in linker sequence, as measured by the difference in the number of bins enriched for/depleted of certain codons across all regions (Table 2), corresponds to enrichment/depletion of codons in sequences selected from simulated sequences for maximal nucleosome exclusion (i.e. linker) potential (rho = 0.62, P = 1.34e-07; see Methods).

*****: see Figure 4 and Table S3.

This rule of thumb can explain the majority of cases where amino acids are depleted from linker regions. Amino acids most strongly enriched in linker (I, L, N, Y), on the other hand, show the strongest and most consistent evidence for biased usage of certain codons (Table 2), and are therefore probably enriched because one or more of their codons is preferentially employed in linker. We tested non-random enrichment/depletion of synonymous codons across OS for each protein abundance bin independently using Fisher's exact test. Of those amino acids (D, F, I, K, L, N, Y) where we find an overall trend for certain codons to be significantly enriched or depleted (Table 2, Table S3, see Methods on how significance was determined), asparagine (N) codons in particular discriminate remarkably well between OSs, with AAT highly enriched in linker sequence (Genomic ratio: AAT/AAC = 1.44, ratio in nucleosome-bound sequence: AAT/AAC = 1.38, ratio in linker: AAT/AAC = 2.5; determined across all bins and regions).

Finally, we compared codon usage in experimentally determined linker sequence with codon usage in sequences selected for maximum nucleosome exclusion potential from simulated sequences (see Methods) and found them to be in good agreement (Figure 4). In particular, all codons consisting entirely of A and T nucleotides are enriched in both simulated and experimentally determined linker sequence. We identify only one codon, GAT, that is not entirely composed of A or T nucleotides. It is interesting to note here that linker elements proximal to nucleosomes can interact with nucleosome remodeling complexes [46],[47] and that Song et al. [48] recently reported recognition motifs of the GATA family of transcription factors to be enriched in nucleosome-free regions at the fission yeast centromere 2, with the binding consensus being centered around the GATA motif.

### Might Linker Be Subject to Alternative Selective Constraints?

The above evidence is consistent with stronger purifying selection acting on linker to maintain correct nucleosome positioning. Could it be, however, that purifying selection is operating, just not as regards nucleosome positioning? We consider two alternatives.

First, might linker sequence be enriched for transcriptional control elements? This seems unlikely for several reasons. Whereas in multicellular eukaryotes it is not unusual for transcription control elements to be located within the open reading frame, transcription regulation in yeast is typically governed by upstream regulatory elements alone [49]. For a handful of genes an effect on expression level upon removal/mutation of specific intra-genic elements has been demonstrated experimentally. However, these elements are mostly located in nucleosome-bound regions (Table S4).

A second possibility is that functional mRNA secondary structure, another cause of sequence conservation and biased composition [1],[4],[50],[51], preferentially maps onto linker sequence. Proposing such a small-scale spatial bias is not unreasonable. We know that nucleosomes are regularly positioned around the promoter, which is also the pivot around which secondary structure facilitating translation initiation is organized [52]. As a result, 5′ regions in yeast are enriched for strong local secondary structures vis-à-vis the remainder of the CDS [51].

Might it be that linker regions and functional secondary structure spatially overlap so that the signature of elevated conservation is really owing to selection on mRNA secondary structure? We find no evidence for this. The window within which hairpin structures downstream of the start codon have an effect on translation initiation (+12–+18 nt [37],[53],[54]) typically fall within the CDS region occupied by the well-positioned nucleosome downstream of the promoter rather than linker sequence (cf. Figure 1A). We also examined a set of strong local mRNA secondary structures (Supplementary Table 1 in [51]), but found no preferential mapping onto linker sequence (Table S5).

## Discussion

The aim of the present analysis was to elucidate whether selection at the DNA level, specifically on nucleosome organization, has affected the evolution of protein-coding sequence. Controlling for intra-genic biases in nucleosome occupancy and, critically, gene expression, we find linker sequence to evolve more slowly, particularly 5′ where constraints are evident on both synonymous and non-synonymous evolution. This is consistent with nucleosome architecture in this region being essential to control gene expression. We estimate that linker sequence across yeast genes evolves approximately 6% slower than sequence bound by nucleosomes. As linker accounts for less than 10% of total genic sequence (with a regional maximum of ∼15% across 5′ regions), the overall reduction in K_s_ is small (<1%). Note, however, that we almost certainly underestimate the effect of nucleosome positioning concerns on coding sequence evolution. This is because our method of detecting selection is based on differences between OSs. In consequence, if nucleosome-bound sequence were also under selection, as suggested by previous research [26],[55], this would lead to an underestimation of the magnitude of selection.

Even assuming that overall effects are modest, however, the results are nonetheless important for several reasons. First, as nucleosome formation on genic sequence is a universal process, our finding of OS-linked evolutionary patterns across regions and expression levels implies that nucleosome positioning, and thus selection at the DNA level, could affect coding sequence evolution in most if not all other eukaryotes. This potentially has direct implications for estimating the neutral mutation rate from genic regions, although as noted above, the effects are probably weak so unlikely to cause serious errors.

Second, while the overall effects on sequence evolution might be minimal vis-à-vis other determinants of substitution rates, synonymous substitutions might individually be of selective significance. The presence of purifying selection certainly argues that individual synonymous mutations have in the past been weeded out because they introduced sequence-based errors in nucleosome positioning. By implication, and given that nucleosomes are a ubiquitous companion of genic sequence, such mutations might be a novel cause of genetic disease.

**Figure 5:**
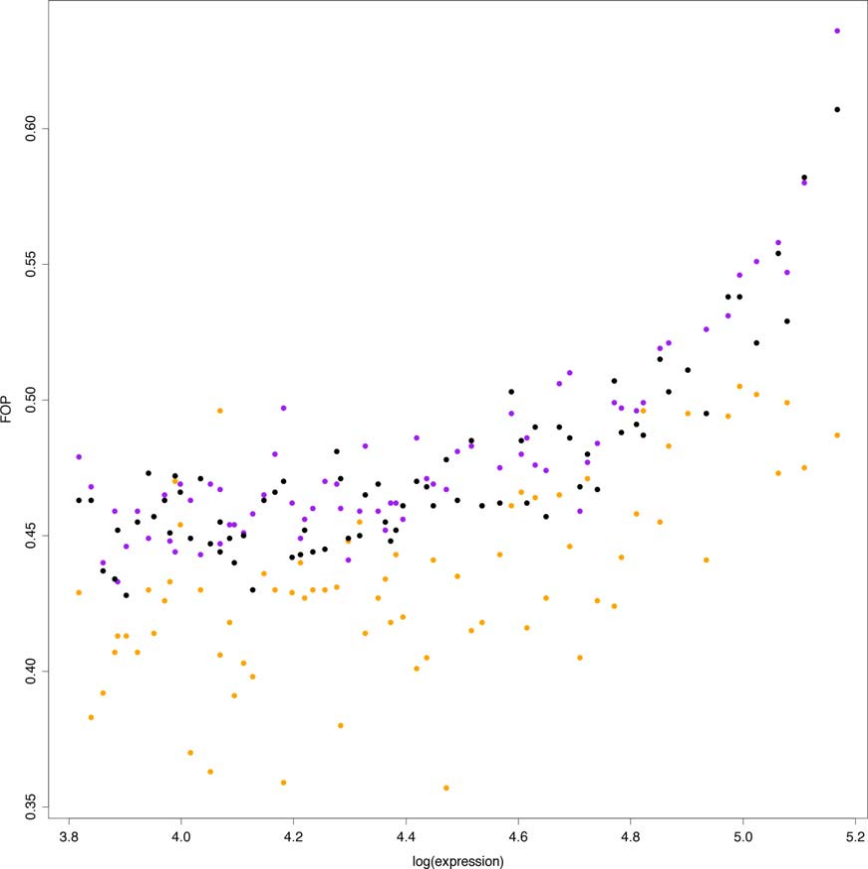
Linker sequence is depleted for translationally optimal codons. The frequency of optimal codons (FOP) as a function of the natural logarithm of protein abundance and nucleosome occupancy across gene cores considering all degenerate amino acids.

Third, these results have an important implication for interpreting local patterns of codon usage. Translationally optimal codons are frequently depleted from linker regions (Table 2). As a result, adaptation for translational efficiency is reduced in linker sequence, as evidenced by a reduced frequency of optimal codons (FOP) (Figure 5; paired t-test for extended core regions: ΔFOP(well-positioned-linker) = 11.20, P<2.2E-16; ΔFOP(fuzzy-linker) = 11.73, P<2.2E-16; ΔFOP(well-positioned-fuzzy) = −3.7, P = 3E-04) and longer runs of translationally non-optimal codons are more likely (Table S6). Previously, runs of non-optimal codons have been considered in the context of selection on translation regulation [56]. Such runs may, for example, induce ribosomal stalling as non-optimal codons tend to be specified by rare tRNAs. This in turn may affect protein folding [57]–[59]. Specification of linker sequence provides a viable alternative hypothesis for a subset of these runs (Table S6).

Finally, the results are consistent with the idea that nucleosome positioning in CDS is in no small part determined by linker-based exclusion signals in contrast to specific nucleosome binding signals, an idea that has recently grown in appreciation [23],[44],[60]. While affinity sequences are more common in coding sequence than expected by chance [55], this signature is relatively weak [26]. If positioning of nucleosomes on CDS is principally achieved by exclusion signals, this is what we expect. Positioning by exclusion might be a particularly beneficial *modus operandi* for coding sequence, as it restricts constraints to a small proportion of an already highly constrained class of sequence.

Note added during production: the observation that linker sequence evolves more slowly has recently been independently made by Washietl et al. [61]

## Methods

### Categorizing Coding Nucleotides by Nucleosome Occupancy

Likely occupancy states (linker, fuzzily, and well-positioned nucleosomes), across the *S. cerevisiae* genome were downloaded from http://chemogenomics.stanford.edu/supplements/03nuc/ (Table S5). *S. cerevisiae* chromosomes were obtained in GenBank format from the Saccharomyces Genome Database (SGD) (ftp://genome-ftp.stanford.edu/pub/yeast/data_download/sequence/genomic_sequence/chromosomes/fasta/archive/genbank_format_20060930.tgz; archived versions from 30/09/2006 to match the data of Lee et al. [23]). Gene models were extracted and filtered so that only genes with a multiple of three nucleotides, proper start and termination codon, no internal stops or ambiguous nucleotides (“n”) were retained. Further, all genes containing introns without consensus splice sites (GT-AG) were eliminated. For each nucleotide in each gene, a likely OS was determined by retrieving all tiling probes (from Lee et al. [23]) containing this nucleotide and determining the dominant call. For example, if covered by 3 probes called as linker, linker, and fuzzy nucleosome, we considered the nucleotide to be in the linker region; regions with 2-probe coverage, where probe calls can be in conflict, were excluded from the analysis, as we had no biological reason to attribute codons to either category. These cases are rare (<0.2% of codons) and thus did not warrant inclusion in a separate category. Only genes in which every nucleotide is covered by at least 2 probes were considered.

### Orthologues

For the filtered set of *S. cerevisiae* genes, orthologues of *S. mikatae* were obtained from SGD (ftp://genome-ftp.stanford.edu/pub/yeast/data_download/sequence/fungal_genomes/S_mikatae/MIT/orf_dna/orf_genomic.fasta.gz). Filters for likely protein-coding capacity were applied as above. The remaining orthologue pairs were aligned at the protein level using MUSCLE (v3.6) after removal of start and stop codons. Alignments with >5% gaps were discarded. Aligned codons for which *S. cerevisiae* OS was consistent across all three nucleotides were concatenated by OS across relevant gene subsets as stated in the Results. K_a_ and K_s_ were calculated using Li's protocol [62].

### Within-Gene Analysis

Analysis of OS-linked differences in sequence evolution were based on a small number of genes (N = 158) with ≥300 coding nucleotides of each major (linker, fuzzy, well-positioned) OS and a sufficient number of degenerate sites to calculate K_s_. Relative rate differentials were calculated as (K_s linker_−K_s well-pos_)/((K_s linker_+K_s well-pos_)/2). The analysis was repeated excluding genes with K_s_ or, more likely, K_a_ = 0. The results remained qualitatively the same (data not shown). Median gene length is markedly longer (median = 2787 nt) than across all yeast genes (median = 1245 nt, Mann-Whitney U test P<2.2E-16), with likely implications for gene function and expression, so that this sample cannot be considered representative.

### Regional Analysis

Genes ≥906 nt without alignment gaps (N = 845, median CDS length = 1473 nt) were considered in the analysis of regional differences. Start and stop codons were trimmed off and terminal (5′ and 3′) and core 100 amino acids concatenated separately. On average, 11010 linker, 54328 fuzzy, and 50780 well-positioned codons were analyzed per region. We chose 100 amino acids as a convenient cut-off as this a) typically captures well-positioned nucleosomes (plus linker) at the start and end of genes (cf. Figure 1A), for which exact positioning is most likely to be of functional significance, and b) analysis of intra-genic substitution variation in prokaryotes [34] suggests that biases extend at least 50 amino acids into the gene. As we do not know what the causes of this variation are or how substantially they affect yeast, a cut-off of 100 amino acids appears a prudent conservative choice. Defining the core as all sequence left after termini have been removed yields qualitatively identical results (data not shown). As the larger amount of sequence available affords a better resolution when the core is defined in this way, we present results for this definition unless otherwise indicated. K_a_ and K_s_ were determined for all aligned concatenates. Significance of differences in evolutionary rates across OSs was tested by repeated random sampling of aligned codon pairs from a region-specific super-concatenate containing all OS concatenates to create 3(OS)×3(regions)×10 000 sequences of the same lengths as the original concatenates. Observing K_a_ (K_s_) values for the original concatenate more than two standard deviations below the mean of the distribution of randomized sequences is taken to be indicative of evolutionary constraint. Concomitant K_a_ (K_s_) values significantly faster than expectation are attributed to the fact that OSs are non-independent. This constraint-guided interpretation is justified because positive selection is expected to be much rarer than purifying selection across the large sample of genes considered here.

### Protein Abundance

Coding sequence concatenated by region and OS was split into expression bins based on protein abundance data from Newman and colleagues [32]. Starting with the gene whose protein was least abundant, sequence from individual genes was allocated to bins of increasing protein abundance. A new bin was generated once the previous bin contained at least 400 codons of the rarest OS, linker. Sequence from any one gene was never split between bins. The results are robust for smaller bins (minimum 250 linker codons) but we decided to prioritize reducing sampling noise for K_a_(K_s_) estimates rather than achieving equal coverage of successive expression ranges. The final bin (highest protein abundance) was discarded because mean average deviation was disproportionally large and the minimum number of codons criterion frequently violated. Differences in evolutionary rates were assessed by analysis of covariance (ANCOVA). OS-specific slopes were shown not to differ significantly, as a prerequisite for assessing the importance of OS as a covariate (Table S2). Average differences in evolutionary rates were quantified by comparing the intercepts of OS-specific slopes (Table S2).

### Codon Usage and Nucleosome Formation Potential

We tested enrichment/depletion of synonymous codons (Table 2) for each protein abundance/region bin independently using Fisher's exact test. At the p<0.05 level we expect N*0.05 bins to show codon skews by chance. With 64 (73, 32) bins in the 5′ (core, 3′) region, we thus expect to see 3.2 (3.65, 1.6) bins with skewed codon usage by chance. Further, there are multiple codons for which significant skews in both directions are observed. This could be owing to both noise in the data and chances of a codon to function as part of linker sequence being dependent on its sequence context. We therefore took a conservative approach to judging whether codon usage is significantly skewed across OS for any one amino acid in that we required A) the difference between numbers of enriched and depleted bins in the core region, for which most data are available, to be 5 or greater and B) the direction of skews not to be inconsistent across regions, e.g. not to find a codon more often enriched than depleted in 5′ regions but more often depleted than enriched in 3′ regions, regardless of whether the relative enrichment in either region was significant on its own.

To evaluate whether codon usage differences across OSs are parsimoniously explained by nucleosome positioning ruled by intrinsic binding affinities, we generated sequences (k = 10 000) of equal length to the region bound by the histone core (147 bp = 49 codons), picking codons at random according to their approximate genomic usage (http://www.kazusa.or.jp/codon/cgi-bin/showcodon.cgi?species=4932). Nucleosome formation potential of these short sequences was scored by assigning a weight to each sequence based on the additive occurrence of all nucleotide k-mers evaluated for their predictiveness in nucleosome positioning by Peckham et al. [44]. Weights corresponded to the receiver operating characteristic (ROC) scores calculated by Peckham et al. [44]. ROC scores reflect the capacity of a k-mer to discriminate between two sets it is differentially represented in, with k-mers of no discriminative power scoring 0.5, a perfect classifier 1.0 (see Peckham et al. [44] and references therein for a more detailed explanation). Overlapping and embedded k-mers were scored as in the following example: 4-mer AAAA was assigned 4× the score for “A”, 3× the score for “AA”, 2× the score for “AAA”, and once the score for the full motif “AAAA”. The overall score was divided by the number of motifs detected. Cross-validation with an alternative algorithm [63] suggests that this approach does, in fact, recover sequences with high and low nucleosome formation potential (Figure S1). Codon usage was compared between the highest and lowest scoring 5% of sequences using a chi-square test. Chi-square cell values were chosen as an approximate measure of codon bias for individual codons (Figure 4).

Codon usage bias towards translationally optimal codons was calculated as the frequency of optimal codons (FOP) [64] using codonw (J.F. Peden) with *S. cerevisiae* default parameters.

SNP analysis is based on data from the Saccharomyces Genome Resequencing Project available at http://www.sanger.ac.uk/Teams/Team71/durbin/sgrp/index.shtml.

Table S7 contains gene names for all *S. cerevisiae* genes used for each major analysis, together with identifiers for orthologous *S. mikatae* ORFs (if applicable). Custom scripts, for example to map nucleosome calls onto coding sequence, are available on request from the authors.

## Supporting Information

Figure S1 Cross-validation of Peckham method. Highest- and lowest-scoring 5% of simulated 49-codon sequences (Materials and Methods) were alternately concatenated (highest-lowest-second highest-second lowest-…) and nucleosome formation potential for the concatenated sequence calculated using RECON [63]. RECON classifies the 49-codon sequences in a fashion consistent with the method derived from the study of Peckham et al. [44]. This is evident from a pattern of oscillation of progressively decreasing amplitude of which the first (left) and last (right) 20*49*3 = 2940 nt are shown.
(5.82 MB TIF)

Table S1 Comparing evolutionary rates across occupancy states for real and randomized concatenates.
(0.02 MB PDF)

Table S2 ANCOVA testing for influence of nucleosome occupancy on evolutionary rates.
(0.04 MB XLS)

Table S3 Fisher's exact tests for biased amino acid usage by amino acid, region, binning protocol, and occupancy state.
(0.03 MB PDF)

Table S4 Nucleosome occupancy at putative intragenic transcriptional regulator elements.
(0.06 MB PDF)

Table S5 Nucleosome occupancy at regions of extremely strong local secondary structure.
(0.04 MB XLS)

Table S6 Runs of unpreferred codons in relation to linker sequence.
(0.01 MB TXT)

Table S7 ORFs used in different analyses.
(0.12 MB TXT)
