# Supplementary figures and images for: The Impact of the Nucleosome Code on Protein-Coding Sequence Evolution in Yeast
Source: PLoS Genet. 2008 Nov 7;4(11):e1000250. doi: 10.1371/journal.pgen.1000250 (PMC2570795; doi:10.1371/journal.pgen.1000250)

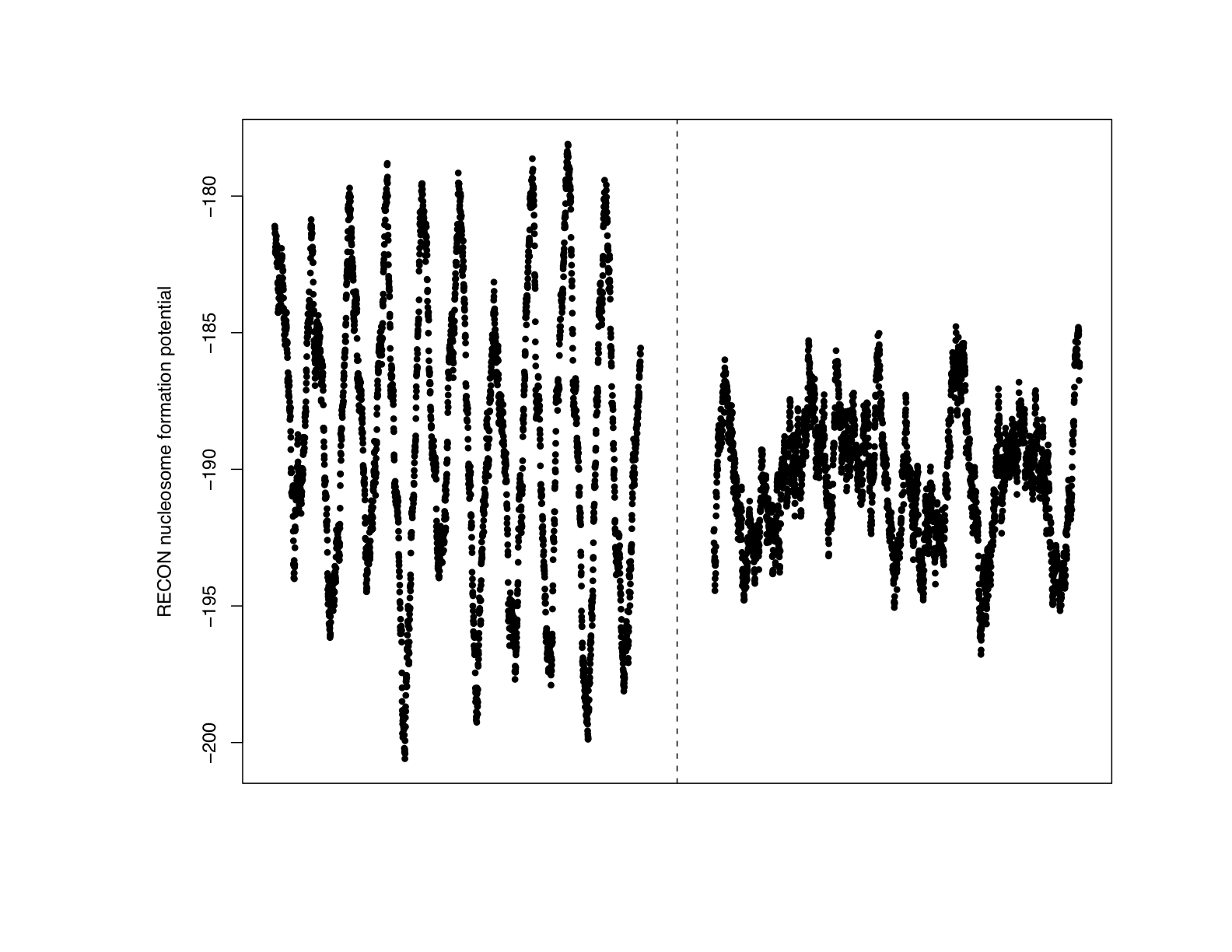

Supplement: Figure S1 — Cross-validation of Peckham method. Highest- and lowest-scoring 5% of simulated 49-codon sequences (Materials and Methods) were alternately concatenated (highest-lowest-second highest-second lowest-…) and nucleosome formation potential for the concatenated sequence calculated using RECON [63]. RECON classifies the 49-codon sequences in a fashion consistent with the method derived from the study of Peckham et al. [44]. This is evident from a pattern of oscillation of progressively decreasing amplitude of which the first (left) and last (right) 20*49*3 = 2940 nt are shown. (5.82 MB TIF) [file pgen.1000250.s001.tif]
