# Supplementary material for: The Impact of the Nucleosome Code on Protein-Coding Sequence Evolution in Yeast
Source: PLoS Genet. 2008 Nov 7;4(11):e1000250. doi: 10.1371/journal.pgen.1000250 (PMC2570795; doi:10.1371/journal.pgen.1000250)
Supplement: Table S1 — Comparing evolutionary rates across occupancy states for real and randomized concatenates. (0.02 MB PDF) [file pgen.1000250.s002.pdf]

Supplementary Table 1

Evolutionary rates of observed concatenated sequence (obs) and number of 10000 randomized (ran) concatenates, that show a Ka (Ks) ≤ or ≥ than the observed value
also see Figures 1B and C

	Ka linker obs	Ka ran ≥ obs	as P value	Ks linker obs	Ks ran ≤ obs	as P value	Ka fuzzy obs	Ka ran ≤ obs	as P value	Ks fuzzy obs	Ks ran ≥ obs	as P value	Ka well-positio	Ka ran ≥ obs	as P value	Ks well-positio	Ks ran ≥ obs	as P value
5'	0.0666	1365	0.1365	0.4833	0	0	0.0539	0	0	0.5152	7108	0.7108	0.0719	0	0	0.5297	1	1.00E-04
core	0.0517	36	0.0036	0.5637	2132	0.2132	0.042	0	0	0.5741	6294	0.6294	0.0518	0	0	0.5786	2425	0.2425
3'	0.0595	176	0.0176	0.5126	136	0.0136	0.0506	0	0	0.5329	9763	0.9763	0.0605	0	0	0.5527	5	5.00E-04
