# Supplementary material for: The Impact of the Nucleosome Code on Protein-Coding Sequence Evolution in Yeast
Source: PLoS Genet. 2008 Nov 7;4(11):e1000250. doi: 10.1371/journal.pgen.1000250 (PMC2570795; doi:10.1371/journal.pgen.1000250)
Supplement: Table S3 — Fisher's exact tests for biased amino acid usage by amino acid, region, binning protocol, and occupancy state. (0.03 MB PDF) [file pgen.1000250.s004.pdf]

Supplementary Table 3 - Fisher's exact tests for biased amino acid usage by amino acid, region, binning protocol, and occupancy state

Amino acid	Region	Binning protocol*	Occupancy state	P value
A	5'	400	linker	2.16E-10
C	5'	400	linker	0.3338597
D	5'	400	linker	0.032623408
E	5'	400	linker	0.050452661
F	5'	400	linker	0.375561337
G	5'	400	linker	2.92E-10
H	5'	400	linker	0.45183646
I	5'	400	linker	4.38E-11
K	5'	400	linker	0.000350896
L	5'	400	linker	0.103435637
M	5'	400	linker	0.200304053
N	5'	400	linker	1.79E-09
P	5'	400	linker	1.12E-07
Q	5'	400	linker	7.21E-05
R	5'	400	linker	0.021607935
S	5'	400	linker	8.05E-06
T	5'	400	linker	0.001222993
V	5'	400	linker	0.004250482
W	5'	400	linker	0.023587816
Y	5'	400	linker	6.86E-10
A	5'	400	fuzzy	0.000109408
C	5'	400	fuzzy	0.147660469
D	5'	400	fuzzy	0.251420036
E	5'	400	fuzzy	0.536182594
F	5'	400	fuzzy	0.067393968
G	5'	400	fuzzy	0.000324973
H	5'	400	fuzzy	0.463996607
I	5'	400	fuzzy	1.03E-06
K	5'	400	fuzzy	2.96E-05
L	5'	400	fuzzy	0.039742682
M	5'	400	fuzzy	0.690698267
N	5'	400	fuzzy	0.000324973
P	5'	400	fuzzy	0.000899059
Q	5'	400	fuzzy	0.182152347
R	5'	400	fuzzy	0.191074464
S	5'	400	fuzzy	0.065415559
T	5'	400	fuzzy	0.072558151
V	5'	400	fuzzy	0.314189796
W	5'	400	fuzzy	0.354331972
Y	5'	400	fuzzy	1.71E-06
A	5'	400	well-positioned	0.033730166
C	5'	400	well-positioned	0.981326206
D	5'	400	well-positioned	0.685775944
E	5'	400	well-positioned	0.666218223
F	5'	400	well-positioned	0.476329018
G	5'	400	well-positioned	0.171438973
H	5'	400	well-positioned	0.859336471
I	5'	400	well-positioned	0.200314117
K	5'	400	well-positioned	0.447833544
L	5'	400	well-positioned	0.35781743
M	5'	400	well-positioned	0.085063638
N	5'	400	well-positioned	0.954669631
P	5'	400	well-positioned	0.812341512
Q	5'	400	well-positioned	0.651696551
R	5'	400	well-positioned	0.032625839
S	5'	400	well-positioned	0.368402697
T	5'	400	well-positioned	0.735574965
V	5'	400	well-positioned	0.476329018
W	5'	400	well-positioned	0.700584538
Y	5'	400	well-positioned	0.567468826
A	Core	400	linker	3.10E-13
C	Core	400	linker	0.113350577
D	Core	400	linker	0.194483922
E	Core	400	linker	0.00012439
F	Core	400	linker	0.018901409
G	Core	400	linker	2.59E-12
H	Core	400	linker	0.563772845
I	Core	400	linker	1.75E-13
K	Core	400	linker	1.55E-06
L	Core	400	linker	7.55E-05
M	Core	400	linker	0.130576195
N	Core	400	linker	1.48E-13
P	Core	400	linker	5.22E-12
Q	Core	400	linker	1.24E-06
R	Core	400	linker	5.75E-06

*see Material and Methods

S	Core	400	linker	7.56E-11
T	Core	400	linker	2.01E-10
V	Core	400	linker	1.75E-12
W	Core	400	linker	0.000113621
Y	Core	400	linker	4.85E-13
A	Core	400	fuzzy	0.582487014
C	Core	400	fuzzy	0.895030686
D	Core	400	fuzzy	0.916809417
E	Core	400	fuzzy	0.873330794
F	Core	400	fuzzy	0.206072902
G	Core	400	fuzzy	0.916809417
H	Core	400	fuzzy	0.877663693
I	Core	400	fuzzy	3.27E-07
K	Core	400	fuzzy	0.037198958
L	Core	400	fuzzy	0.089367276
M	Core	400	fuzzy	0.70036417
N	Core	400	fuzzy	0.509442052
P	Core	400	fuzzy	0.002834181
Q	Core	400	fuzzy	0.057150974
R	Core	400	fuzzy	0.0371986
S	Core	400	fuzzy	0.003899033
T	Core	400	fuzzy	0.010916249
V	Core	400	fuzzy	0.276367336
W	Core	400	fuzzy	0.672067193
Y	Core	400	fuzzy	4.21E-05
A	Core	400	well-positioned	1.72E-05
C	Core	400	well-positioned	0.488502824
D	Core	400	well-positioned	0.745668675
E	Core	400	well-positioned	0.733217657
F	Core	400	well-positioned	0.145157143
G	Core	400	well-positioned	0.00169465
H	Core	400	well-positioned	0.593851582
I	Core	400	well-positioned	0.013994383
K	Core	400	well-positioned	0.036701307
L	Core	400	well-positioned	0.020641896
M	Core	400	well-positioned	0.66806196
N	Core	400	well-positioned	0.006185489
P	Core	400	well-positioned	0.005687558
Q	Core	400	well-positioned	0.133398814
R	Core	400	well-positioned	0.181579963
S	Core	400	well-positioned	0.929907756
T	Core	400	well-positioned	0.34717546
V	Core	400	well-positioned	0.076691658
W	Core	400	well-positioned	0.582487014
Y	Core	400	well-positioned	0.196382889
A	3'	400	linker	1.50E-05
C	3'	400	linker	0.646856147
D	3'	400	linker	0.815173493
E	3'	400	linker	0.010135837
F	3'	400	linker	0.242510489
G	3'	400	linker	4.04E-06
H	3'	400	linker	0.147273034
I	3'	400	linker	8.33E-07
K	3'	400	linker	0.01800732
L	3'	400	linker	9.66E-05
M	3'	400	linker	0.127459564
N	3'	400	linker	2.08E-07
P	3'	400	linker	1.12E-05
Q	3'	400	linker	0.006478708
R	3'	400	linker	0.002527657
S	3'	400	linker	2.02E-05
T	3'	400	linker	0.000296427
V	3'	400	linker	0.065491853
W	3'	400	linker	0.000788635
Y	3'	400	linker	5.28E-06
A	3'	400	fuzzy	0.286517813
C	3'	400	fuzzy	0.789125923
D	3'	400	fuzzy	0.963233216
E	3'	400	fuzzy	0.719023432
F	3'	400	fuzzy	0.037587175
G	3'	400	fuzzy	0.035818879
H	3'	400	fuzzy	0.090818201
I	3'	400	fuzzy	4.89E-05
K	3'	400	fuzzy	0.004412336
L	3'	400	fuzzy	0.171616049
M	3'	400	fuzzy	0.560757357
N	3'	400	fuzzy	0.077048062
P	3'	400	fuzzy	0.106437701

Q	3'	400	fuzzy	0.026594988
R	3'	400	fuzzy	0.278129682
S	3'	400	fuzzy	0.077048062
T	3'	400	fuzzy	0.190094438
V	3'	400	fuzzy	0.488326314
W	3'	400	fuzzy	0.904595608
Y	3'	400	fuzzy	0.002073234
A	3'	400	well-positioned	0.077048062
C	3'	400	well-positioned	0.548343148
D	3'	400	well-positioned	0.560757357
E	3'	400	well-positioned	0.585975122
F	3'	400	well-positioned	0.035818879
G	3'	400	well-positioned	0.037587175
H	3'	400	well-positioned	0.183783332
I	3'	400	well-positioned	0.760850264
K	3'	400	well-positioned	0.488326314
L	3'	400	well-positioned	0.246213506
M	3'	400	well-positioned	0.677999434
N	3'	400	well-positioned	0.090818201
P	3'	400	well-positioned	0.598772789
Q	3'	400	well-positioned	0.154479305
R	3'	400	well-positioned	0.246213506
S	3'	400	well-positioned	0.523917939
T	3'	400	well-positioned	0.875446003
V	3'	400	well-positioned	0.286517813
W	3'	400	well-positioned	0.454044791
Y	3'	400	well-positioned	0.454044791
A	5'	250	linker	5.44E-13
C	5'	250	linker	0.057486833
D	5'	250	linker	0.073031926
E	5'	250	linker	0.091835113
F	5'	250	linker	0.37276451
G	5'	250	linker	1.00E-12
H	5'	250	linker	0.536855359
I	5'	250	linker	4.22E-14
K	5'	250	linker	0.000228361
L	5'	250	linker	0.125462067
M	5'	250	linker	0.073565452
N	5'	250	linker	9.21E-11
P	5'	250	linker	1.72E-08
Q	5'	250	linker	8.29E-05
R	5'	250	linker	0.034591578
S	5'	250	linker	3.26E-06
T	5'	250	linker	0.000300007
V	5'	250	linker	0.002660986
W	5'	250	linker	0.002511684
Y	5'	250	linker	6.75E-11
A	5'	250	fuzzy	9.93E-05
C	5'	250	fuzzy	0.239976744
D	5'	250	fuzzy	0.314996324
E	5'	250	fuzzy	0.794572069
F	5'	250	fuzzy	0.057948328
G	5'	250	fuzzy	2.17E-05
H	5'	250	fuzzy	0.723452787
I	5'	250	fuzzy	4.18E-07
K	5'	250	fuzzy	4.88E-06
L	5'	250	fuzzy	0.070405253
M	5'	250	fuzzy	0.875327871
N	5'	250	fuzzy	0.000219567
P	5'	250	fuzzy	0.001663314
Q	5'	250	fuzzy	0.126292124
R	5'	250	fuzzy	0.070405057
S	5'	250	fuzzy	0.033746842
T	5'	250	fuzzy	0.037861674
V	5'	250	fuzzy	0.326382637
W	5'	250	fuzzy	0.52373767
Y	5'	250	fuzzy	8.63E-07
A	5'	250	well-positioned	0.016388157
C	5'	250	well-positioned	0.94411258
D	5'	250	well-positioned	0.398356545
E	5'	250	well-positioned	0.797147754
F	5'	250	well-positioned	0.572650192
G	5'	250	well-positioned	0.093783783
H	5'	250	well-positioned	0.841252743
I	5'	250	well-positioned	0.266303911
K	5'	250	well-positioned	0.379975289
L	5'	250	well-positioned	0.098453032
M	5'	250	well-positioned	0.019280107

N	5'	250	well-positioned	0.899058331
P	5'	250	well-positioned	0.8517091
Q	5'	250	well-positioned	0.784291274
R	5'	250	well-positioned	0.055350113
S	5'	250	well-positioned	0.536862733
T	5'	250	well-positioned	0.635484345
V	5'	250	well-positioned	0.483291664
W	5'	250	well-positioned	0.713469646
Y	5'	250	well-positioned	0.628359341
A	Core	250	linker	4.41E-19
C	Core	250	linker	0.111409339
D	Core	250	linker	0.134730592
E	Core	250	linker	4.56E-05
F	Core	250	linker	0.017280663
G	Core	250	linker	7.89E-18
H	Core	250	linker	0.515492825
I	Core	250	linker	0
K	Core	250	linker	4.33E-08
L	Core	250	linker	7.82E-06
M	Core	250	linker	0.087677479
N	Core	250	linker	0
P	Core	250	linker	3.52E-14
Q	Core	250	linker	1.74E-07
R	Core	250	linker	3.94E-06
S	Core	250	linker	1.02E-12
T	Core	250	linker	1.08E-13
V	Core	250	linker	3.35E-16
W	Core	250	linker	6.02E-06
Y	Core	250	linker	0
A	Core	250	fuzzy	0.445226827
C	Core	250	fuzzy	0.694313466
D	Core	250	fuzzy	0.921170954
E	Core	250	fuzzy	0.810075524
F	Core	250	fuzzy	0.246361599
G	Core	250	fuzzy	0.845322274
H	Core	250	fuzzy	0.77737651
I	Core	250	fuzzy	1.59E-07
K	Core	250	fuzzy	0.021204284
L	Core	250	fuzzy	0.167657807
M	Core	250	fuzzy	0.786060369
N	Core	250	fuzzy	0.380762326
P	Core	250	fuzzy	0.003556378
Q	Core	250	fuzzy	0.173836941
R	Core	250	fuzzy	0.054526683
S	Core	250	fuzzy	0.002370106
T	Core	250	fuzzy	0.060852855
V	Core	250	fuzzy	0.275108088
W	Core	250	fuzzy	0.950401415
Y	Core	250	fuzzy	3.70E-05
A	Core	250	well-positioned	3.21E-06
C	Core	250	well-positioned	0.426905293
D	Core	250	well-positioned	0.52466957
E	Core	250	well-positioned	0.474404529
F	Core	250	well-positioned	0.076265542
G	Core	250	well-positioned	0.001384872
H	Core	250	well-positioned	0.77737651
I	Core	250	well-positioned	0.012340854
K	Core	250	well-positioned	0.045003336
L	Core	250	well-positioned	0.008340646
M	Core	250	well-positioned	0.912196504
N	Core	250	well-positioned	0.006363534
P	Core	250	well-positioned	0.004218737
Q	Core	250	well-positioned	0.048115115
R	Core	250	well-positioned	0.095845772
S	Core	250	well-positioned	0.948150041
T	Core	250	well-positioned	0.132536491
V	Core	250	well-positioned	0.055601423
W	Core	250	well-positioned	0.379227588
Y	Core	250	well-positioned	0.205271976
A	3'	250	linker	5.18E-07
C	3'	250	linker	0.76062938
D	3'	250	linker	0.936494426
E	3'	250	linker	0.017491363
F	3'	250	linker	0.178585826
G	3'	250	linker	1.02E-06
H	3'	250	linker	0.473316195
I	3'	250	linker	2.88E-09
K	3'	250	linker	0.012820252

L	3'	250	linker	9.89E-06
M	3'	250	linker	0.224776764
N	3'	250	linker	3.73E-06
P	3'	250	linker	7.78E-05
Q	3'	250	linker	0.002502881
R	3'	250	linker	0.000952412
S	3'	250	linker	0.000102149
T	3'	250	linker	0.000402343
V	3'	250	linker	0.036171628
W	3'	250	linker	0.000446522
Y	3'	250	linker	1.65E-07
A	3'	250	fuzzy	0.169686172
C	3'	250	fuzzy	0.683460303
D	3'	250	fuzzy	0.676591167
E	3'	250	fuzzy	0.973828389
F	3'	250	fuzzy	0.021916456
G	3'	250	fuzzy	0.048482487
H	3'	250	fuzzy	0.107933865
I	3'	250	fuzzy	1.46E-05
K	3'	250	fuzzy	0.002831622
L	3'	250	fuzzy	0.147560948
M	3'	250	fuzzy	0.514752205
N	3'	250	fuzzy	0.032968745
P	3'	250	fuzzy	0.072650688
Q	3'	250	fuzzy	0.158331096
R	3'	250	fuzzy	0.118598745
S	3'	250	fuzzy	0.036173673
T	3'	250	fuzzy	0.158331096
V	3'	250	fuzzy	0.55797975
W	3'	250	fuzzy	0.936496166
Y	3'	250	fuzzy	0.001461144
A	3'	250	well-positioned	0.02302222
C	3'	250	well-positioned	0.396271613
D	3'	250	well-positioned	0.656146557
E	3'	250	well-positioned	0.49677275
F	3'	250	well-positioned	0.322711701
G	3'	250	well-positioned	0.027943549
H	3'	250	well-positioned	0.232041311
I	3'	250	well-positioned	0.811087434
K	3'	250	well-positioned	0.350995568
L	3'	250	well-positioned	0.243211732
M	3'	250	well-positioned	0.796584157
N	3'	250	well-positioned	0.096152503
P	3'	250	well-positioned	0.642657339
Q	3'	250	well-positioned	0.118598745
R	3'	250	well-positioned	0.412111874
S	3'	250	well-positioned	0.467566821
T	3'	250	well-positioned	0.767790819
V	3'	250	well-positioned	0.197447189
W	3'	250	well-positioned	0.309143366
Y	3'	250	well-positioned	0.526921413
