# Supplementary material for: The Impact of the Nucleosome Code on Protein-Coding Sequence Evolution in Yeast
Source: PLoS Genet. 2008 Nov 7;4(11):e1000250. doi: 10.1371/journal.pgen.1000250 (PMC2570795; doi:10.1371/journal.pgen.1000250)
Supplement: Table S4 — Nucleosome occupancy at putative intragenic transcriptional regulator elements. (0.06 MB PDF) [file pgen.1000250.s005.pdf]

Supplementary Table 4. Nucleosome occupancy at putative intragenic transcriptional regulator elements.

Gene	Location of putative transcriptional enhancer/repressor (nt relative to translational start)	Reported by	Nucleosome occupancy (0=Linker, 1=Fuzzy, 2=Well-positioned; see Material and Methods for other codes)
FAS2 (YPL231W)	+22 to +66 (downstream repressor sequence)	[1]	224444000003333111111111111111111111111111111
HXK2 (YGL253W)	+140 to +163 (downstream repressor sequence 1)	[2]	111111111111111111111111
HXK2 (YGL253W)	+231 to +251 (downstream repressor sequence 2)	[2]	111111111111111111111
LPD1 (YFL018C)	+137 to +191 (downstream activation sequence 1)	[3]	111111111111111111111111111111111111111111111111111111 1
LPD1 (YFL018C)	+291 to +296 (downstream activation sequence 2)	[3]	222222
LPD1 (YFL018C)	+288 to +313 (downstream repressor sequence 1)	[3]	22222222222222222222222222
LPD1 (YFL018C)	+406 (downstream repressor sequence 2)	[3]	2

PGK (YCR012W)	+37 to +236 (downstream activator sequence)	[4]	111111111111111111111111111111111111111111111111111111<br>111111111111111111111111111111111111111111111111111111<br>111111111111111111111111111111111111111111111111111111<br>11111111111111111111111111111111111111

References

1. Wenz P, Schwank S, Hoja U, Schuller HJ (2001) A downstream regulatory element located within the coding sequence mediates autoregulated expression of the yeast fatty acid synthase gene FAS2 by the FAS1 gene product. Nucleic Acids Res 29: 4625-4632.
2. Herrero P, Ramirez M, Martinez-Campa C, Moreno F (1996) Identification and characterisation of two transcriptional repressor elements within the coding sequence of the Saccharomyces cerevisiae HXK2 gene. Nucleic Acids Res 24: 1822-1828.
3. Sinclair DA, Kornfeld GD, Dawes IW (1994) Yeast intragenic transcriptional control: activation and repression sites within the coding region of the Saccharomyces cerevisiae LPD1 gene. Mol Cell Biol 14: 214-225.
4. Mellor J, Dobson MJ, Kingsman AJ, Kingsman SM (1987) A transcriptional activator is located in the coding region of the yeast PGK gene. Nucleic Acids Res 15: 6243-6259.
